# Comorbidity and concomitant medication use in an integrase strand transfer inhibitor naïve cohort on first-line dolutegravir-based antiretroviral therapy

**DOI:** 10.11604/pamj.2024.47.137.40726

**Published:** 2024-03-25

**Authors:** Nivriti Hurbans, Panjasaram Naidoo

**Affiliations:** 1 Discipline of Pharmaceutical Sciences, School of Health Sciences, College of Health Science, University of KwaZulu-Natal, Westville, Durban, South Africa,; 2 South African Medical Research Council, HIV and Other Infectious Diseases Research Unit, Durban, South Africa

**Keywords:** Dolutegravir, comorbidities, concomitant medication, co-infection, drug interaction, HIV/AIDS

## Abstract

**Introduction:**

people living with HIV/AIDS using antiretroviral therapy sometimes present with comorbid conditions or co-infections. This could lead to an increased risk of drug interactions due to the concomitant use of drugs. The aim of the study was to explore the overall impact of dolutegravir on such comorbidities and the effect of concomitant medication on the safety and efficacy of dolutegravir.

**Methods:**

data was collected using a survey questionnaire and a retrospective review of medical records of a prospective study sample. Medical records were retrospectively reviewed for up to 12 months after dolutegravir initiation. Concomitantly used drugs and supplements that were identified to have a potential interaction with dolutegravir were further characterized. Descriptive and summary statistics were used to describe the data, t-tests were performed on blood glucose levels and cross-tabulations were done on some variables.

**Results:**

of the 461 participants enrolled into the study, 172 (37.3%) and 54 (11.7%) experienced comorbidity and coinfection respectively. More than 50% of the participants used concomitant medicines. Metformin use led to increased blood glucose levels (p=0.009); participants on rifampicin (n=8) received an additional daily dose of dolutegravir. Virological outcomes in participants on sodium valproate (n=2) and St John´s wort (n=1) did not show safety concerns, whilst 3 dolutegravir discontinuations were observed in participants using supplements and antacids containing divalent cations.

**Conclusion:**

even though dolutegravir was safe and effective in the study population, with possible drug interactions leading to treatment discontinuations in only 3(0.7%) participants, further investigation into dolutegravir-induced hyperglycemia needs investigation.

## Introduction

Antiretroviral therapy (ART) has transformed human immunodeficiency virus (HIV)/acquired immunodeficiency syndrome (AIDS) into a chronic illness, where infected individuals have a lifelong dependency on ART [1]. The long-term use of ART and the prevalence of co-morbidities and co-infections could pose an increased risk of drug interactions with ART and non-ART drugs [2,3]. Dolutegravir (DTG), an integrase strand transfer inhibitor (INSTI) has the potential to revolutionize HIV treatment and is recommended by the World Health Organization in first-line ART regimens, as the preferred treatment choice. It is therefore anticipated that millions of people will use this drug especially in low- and middle-income countries [4].

Comorbidities vary in different communities and are associated with long-term use of drugs. In general, there is a high prevalence of hypertension in the South African general population [5]. The high prevalence of comorbidities presents challenges in individuals receiving ART [6]. An understanding of concomitant medication used in the treatment of comorbidities and co-infections in a study population is important to be able to evaluate the risks associated and potential drug interactions. Co-infections are associated with the short-term use of drugs which can be a few days to a few weeks, except for tuberculosis and certain fungal infections which often require a treatment duration of up to 6 months. Other drugs for consideration are those used to treat side effects and other clinically insignificant conditions.

An increased prevalence of hypertension and other comorbidities has been observed in HIV-positive individuals [7]. The duration of HIV infection, viral load (VL), CD4 count [8], age, body mass index, and DTG use [9] are some predictors of hypertension amongst people living with HIV and AIDS. There has been increased use of DTG in sub-Saharan Africa, but limited data on its association with hypertension [9]. Integrase strand transfer inhibitors (INSTI) have been associated with hyperglycaemia, ranging from moderate to severe [10-12]. Hailu *et al*. have also reported on the increased incidence of diabetes after the use of DTG-based ART [13].

Drug interactions between ART and non-ART drugs of clinical relevance are a regular phenomenon and can lead to toxicity or reduced efficacy when co-administered. ART drugs can induce or inhibit drug-metabolizing enzymes or drug transporters [14]. Dolutegravir (DTG) is metabolized via uridine diphosphate glucuronyltransferase (UGT) 1A1 (major) and cytochrome P450 (minor) pathways [15,16]. Whilst DTG has the potential to interact with ART and non-ART drugs, in most instances, this interaction is clinically insignificant [17,18]. However, significant pharmacokinetic and pharmacodynamic interactions between DTG with other drugs do exist. These include metformin [19], rifampicin [20], sodium valproate [2], and cation-containing formulations [21,22]. Data on the clinical significance of these interactions is still inadequate.

Therefore, as countries roll out DTG-based ART, key questions that need to be answered includes: how would DTG interact with concurrently administered drugs? Would any potential drug interaction reduce the benefit of the transition to the DTG-based regimen? Is there a link between DTG use and the onset of hypertension and diabetes? The aim of this study was to determine the extent of comorbidity and concomitant medicine use in a cohort of people living with HIV/AIDS and ascertain whether any drugs used will have an overall impact on the safety and efficacy of DTG-based ART.

## Methods

**Study design and setting:** two study designs were used to collect the data. The first was a cross-sectional survey, followed by a longitudinal retrospective review of medical records. The study was conducted at three public health facilities in the eThekwini Metropolitan of KwaZulu Natal. Convenience sampling was used to select the health facilities based on the following criterion: a specialized HIV clinic, at least 20 ART initiations per month, and a separate filing room for these files.

**Study population:** the study population comprised of all HIV-positive individuals, 18 years and over who were on first-line DTG containing ART for between 4-8 months and were willing to provide written informed consent. A sample size of 415 participants was calculated. Study participants were recruited from the waiting areas of the health facilities.

**Data collection tools and procedures:** data collection tools included a questionnaire and a medical record review tool. The questionnaire comprised of open and closed-ended questions on socio-demographics, co-morbidity, co-infection, and use of concomitant medication. The questionnaire was designed to be self-administered; however, assistance was provided upon request by the study participants. All questionnaires were available in English and IsiZulu. Data collectors assisted with the administration of the questionnaires and translation of information that may have been recorded in Isizulu. All questionnaires were administered between October 2020 and February 2021. The medical record review tool included the collection of data on clinical investigations, comorbidity, concomitant medication, and relevant medical history. All data from the medical records was evaluated from DTG initiation and up to 12 months thereafter if the participant was still on the DTG-based ART. Medical records were reviewed between October 2020 and January 2022.

In this study the following definitions were applied: comorbidity was any chronic condition that was simultaneously present with HIV/AIDS; co-infection was the presence of any bacterial, viral, fungal or parasitic infection; and concomitant medication was any non-ART medication. We assessed comorbidity and all drugs used by study participants, either by self-report or recorded in the hospital/clinic medical records. Potential drug interactions were identified using the DTG (Tivicay) package insert [23], EM Guidance website [24], and HIV drug interactions website [25].

**Data analysis:** all data for the study was captured onto REDCap and thereafter exported into Statistical Package for Social Sciences Version 28, where further analyses were undertaken. Descriptive and summary statistics were used to describe the data. Numbers and percentages were used to describe categorical variables. A t-test was performed to determine the impact of DTG on blood glucose levels. Cross-tabulations were done on some variables.

**Ethical consideration:** the study was approved by the University of KwaZulu-Natal Biomedical Research Ethics Committee with Ethics approval number BREC 442/19.

## Results

A total of 461 participants were enrolled in the study. The age of study participants was categorized as follows: 18-30 years, n=33 (7.2%); 31-40 years, n=108 (23.4%); 41-50 years, n=160 (34.7%); 51-60 years, n=123 (26.7%) and more than 60 years, n=37 (8.0%). A total of 227 (49.2%) males and 234 (50.8%) females were enrolled with 431 (93.5%) being ART experienced at the time of enrolment.

**Comorbidity and co-infection:** Table 1 summarizes the comorbid conditions and co-infections experienced by the study participants. Comorbid conditions were present in 172 (37.3%) of the study participants. Of these participants, 113 (65.7%) participants only had 1 comorbid condition, 40 (23.3%) had 2 comorbid conditions, 17 (9.9%) had 3 comorbid conditions and 1 (0.6%) had 4 comorbid conditions. Of the 124 participants that were hypertensive, 111 (89.5%) were hypertensive prior to starting DTG-based ART, 10 (8.1%) were noted to be hypertensive by self-report, and 3 (2.4%) became hypertensive after starting DTG-based ART. Of the 3 participants who became hypertensive after starting DTG-based ART, a cross-tabulation did not show any association between the onset of hypertension and age, prior ART usage, VL, or weight. A total of 30 participants were diabetic with only 2 incidents of diagnosis after DTG initiation. A cross-tabulation did not show any relationship between increased blood glucose levels with VL, CD4 counts, weight, clinical staging, metformin dose, age, or gender. A total of 54 (11.7%) participants were co-infected with either a bacterial, viral, fungal, or parasitic infection and had received treatment for such conditions.

**Table 1:** summary of comorbidities and co-infections present in study participants

Variable	Number	Percent
**Comorbidity**		
Hypertension	124	26.9
Diabetes	30	6.5
Hypercholesterolemia	36	7.8
Arthritis	34	7.4
Asthma	15	3.3
Epilepsy	6	1.3
Heart failure	1	0.2
Hypothyroidism	3	0.7
Gout	2	0.4
Circulation disorders	2	0.4
**Co-infection**		
Tuberculosis	7	1.5
Bacterial infections	18	3.9
Fungal infections	12	2.6
Parasitic infections	1	0.2
Sexually transmitted infections	17	3.7

**Concomitant medication:** all systemic drugs used by study participants, either by self-report or recorded in the hospital/clinic medical records are summarized in Table 2. Combination drugs have been disaggregated and listed separately. Drugs administered topically included antifungal creams, steroidal creams, suppositories, antiseptic ointments, ophthalmic ointments and nasal sprays, and inhalers. Table 3 summarizes the virological outcomes of study participants who received drugs that were identified to potentially interact with DTG. Of the diabetic participants, 24 (80%) were treated with metformin. In diabetic participants treated with metformin, the dose varied between 500 mg to ≥1500 mg daily. Baseline blood glucose levels were only available for 10 of these participants whilst blood glucose levels were available for 12 participants after the start of DTG. Where multiple follow-up values were available, the latest blood glucose value was used. The mean blood glucose levels were 7.2 ± 1.6 (n=10) and 14.1 ± 6.6 (n=12) for baseline and after DTG initiation respectively.

**Table 2:** summary of systemic drugs used by study participants

Condition	Drug
Hypertension	Amlodipine, hydrochlorothiazide, atenolol, enalapril, hydralazine, acetylsalicylic acid
Diabetes	Metformin, insulin, glibenclamide
Hypercholesterolemia	Simvastatin, atorvastatin, bezafibrate
Arthritis	Ibuprofen
Asthma	Salbutamol inhaler
Epilepsy	Lamotrigine, sodium valproate
Heart failure	Furosemide
Hypothyroidism	Levothyroxine
Gout	Allopurinol
Circulation disorders	Warfarin
Tuberculosis	Rifampicin, isoniazid, pyrazinamide, ethambutol; trimethoprim-sulfamethoxazole
Bacterial infections	Amoxicillin, clavulanic acid, ciprofloxacin
Fungal infections	Fluconazole
Parasitic infections	Albendazole
Sexually transmitted infections	Azithromycin, metronidazole, ceftriaxone, doxycycline, benzathine penicillin
Gastrointestinal disorders	Lansoprazole, aluminium hydroxide/magnesium trisilicate, loperamide, lactulose, hyoscine butylbromide
Analgesics	Paracetamol, ibuprofen, tramadol
Vitamins/supplements	Ferrous sulphate, folic acid, vitamin B 12, calcium gluconate, pyridoxine, multivitamins, vitamin B complex
Miscellaneous	Chlorpheniramine, cetirizine, amitriptyline, St. John’s wort *hypericum perforatum*)

**Table 3:** virological outcomes of study participants with concomitant use of drugs that have the potential to lower dolutegravir (DTG) concentrations

Viral load (copies/ml)	Rifampicin (n=7)	Sodium valproate (n=2)	St John’s wort (n=1)	Ca, Mg, Fe, and Al supplements and/or antacids (n=136)
**Baseline**				
<50	4	2	1	114
50-999	-	-	-	10
≥1000	1	-	-	1
Not available	2	-	-	11
**After DTG**				
<50	6	2	1	118
50-999	-	-	-	8
≥1000	1	-	-	2
Not available	-	-	-	8

Ca: calcium; Mg: magnesium; Fe: iron; Al: aluminium; DTG: dolutegravir

A t-test was done to determine whether DTG had an impact on metformin levels by measuring the effect on blood glucose levels. Only 6 pairs of results were used in the t-test analysis due to missing data, p=0.009. Only one participant who was on metformin used a magnesium-containing medicine/supplement. Table 4 summarizes the method of use of multivitamins, calcium, and iron supplements as per self-report. A further 7 (1.5%) participants used herbal medicines and 24 (5.2%) used traditional medicines.

**Table 4:** supplement use by self-report

Supplement use by self-report	Number (%)
**Calcium (n=40)**	
6 hours after ARVs	20 (50.0)
2 hours before ARVs	10 (25.0)
With ARV’s	4 (10.0)
Other (when necessary, n=2; in the mornings n=3)	6 (15.0)
**Iron (n=16)**	
6 hours after ARVs	3 (18.8)
2 hours before ARVs	5 (31.2)
With ARV’s	3 (18.8)
Other (after ARVs n=1; twice daily n=2; thrice daily n=1; after food n=1)	5 (31.2)
**Multivitamins (n=58)**	
6 hours after ARVs	25 (43.1)
2 hours before ARVs	10 (17.2)
With ARV’s	18 (31.1)
Other (in the mornings n=2; anytime n=2, one hour after ARVs n=1)	5 (8.6)

ARVs: antiretrovirals

## Discussion

The results of this study presented a fair overview of the concomitant medicines used by the study participants. Additionally, it also provided information on the prevalence and types of comorbidities and co-infections that were present in this cohort of patients.

Hypertension was found to be the most common comorbidity experienced by 26.9% of the study participants. Diabetes and epilepsy were found to be present in 6.5% and 1.3% of the study population respectively. Bacterial infections excluding tuberculosis, were the most commonly recorded co-infection, experienced by 3.9% of participants followed by sexually transmitted infections experienced by 3.7% of study participants.

Despite hypertension being experienced by more than a quarter of the study population, none of the drugs as recorded in the medical records used for its treatment (Table 2) have been documented to interact with DTG [23-25]. Being the most reported comorbidity, we further investigated whether DTG use was associated with an increased incidence of hypertension. Of the 3 participants who became hypertensive after starting the DTG-based ART, no association could be found between the onset of hypertension with age, prior ART usage, VL, and weight gain. Whilst studies have shown a causal relationship between DTG use and increased risk of hypertension [9,26-29], our study in agreement with other studies [30,31], were unable to show similar outcomes.

The co-administration of DTG and metformin has been known to result in increased metformin levels due to a decrease in its clearance, owing to inhibition of the organic cation transporter 2 pathway [19]. This occurs in a dose-dependent manner, where larger doses of metformin leads to a more attenuated effect [19]. For this reason, it is suggested that daily metformin doses should not exceed 1000 mg when administered together with DTG [32]. Decreased clearance of metformin has been documented to cause hypoglycemia and it is therefore crucial to monitor serum blood glucose levels when metformin is co-administered with DTG [33]. In this study, we compared the blood glucose levels of participants on metformin at baseline and after taking DTG-based ART. We noted increased serum blood glucose levels, p=0.009. This finding was not anticipated, and we attempted to find a plausible explanation for this effect. We could not find any causal relationship between increased serum glucose levels and VL, CD4 counts, weight, clinical staging, metformin dose, age or gender. Recently, there have been reports of hyperglycemia in study participants following the use of INSTIs [34,35], whilst other studies have shown that DTG did not have any effect on the glycaemic levels and was well tolerated when used together with metformin [19,36], and limiting the use of DTG in patients at high risk of diabetes was not necessary [37]. We also looked at the incidence of diabetes post-DTG-based ART initiation and found that only 2 (0.4%) participants became diabetic after commencing DTG. The cause of DTG-related hyperglycaemia is poorly understood. One hypothesis is the chelation of magnesium that leads to the inhibition of the release of insulin [29]. We tested this hypothesis in our study and found that only one participant that was on metformin used a magnesium-containing concomitant medicine/supplement. Furthermore, blood glucose levels were not available for this participant after commencing DTG-based. This hypothesis therefore needs further evaluation.

In this study, 7 (1.5%) participants had tuberculosis and received rifampicin-containing tuberculosis treatment, together with an additional dose of 50 mg DTG. The additional dose of 50 mg DTG was administered to these participants as it is known that rifampicin enhances the metabolism of DTG [15], thus by increasing the DTG dose to 50 mg twice daily in the presence of rifampicin provided similar levels to those achieved with a standard dose of 50 mg once daily [38,39]. After starting DTG, 6 (85.7%) of these participants had undetectable VLs and only 1 (14.3%) had a VL ≥1000. The baseline VL was not available for this participant so we cannot determine whether this was an increase from baseline. The participant who initially had a VL of ≥1000 at baseline showed an undetectable VL after DTG initiation. Despite 3 of the participants on rifampicin having reported side effects, there were no safety concerns and no treatment-related discontinuations due to DTG. Although rifamycins remain the mainstay of tuberculosis treatment due to their sterilizing ability [40], co-treatment of tuberculosis and HIV can result in drug toxicities and drug interactions [41] of concern. The additional dose of DTG may also be challenging to implement in settings where the incidence of HIV is high as the pill burden and costs increase [42]. The South African national guidelines therefore recommend that efavirenz-based ART should be the preferred treatment option in patients with tuberculosis co-infection [43]. In addition, recent evidence has shown that increasing the dosing complexity and the added burden of an additional dose of DTG in the presence of tuberculosis treatment may lead to decreased adherence and increased loss to follow-up [44]. It has also been shown that viral suppression rates in the presence of tuberculosis treatment were comparable in groups receiving DTG once or twice daily and non-DTG ART regimens [44,45]. Further evidence to support the standard dose of DTG together with rifampicin would be needed before decision-makers can provide further recommendations. Le *et al*. showed that twice daily dosing of DTG with the standard dose of rifampicin was equivalent to once-daily dosing of dolutegravir with rifabutin [46]. Rifabutin can therefore be a suitable alternative to rifampicin to simplify DTG-based ART regimens in individuals co-infected with tuberculosis.

Two of the epileptic participants who were treated with sodium valproate, had undetectable VLs before and after DTG initiation. They however did experience headaches as a common side-effect, but neither discontinued treatment. Whilst sodium valproate is expected to decrease DTG concentrations, the present study showed that neither of the participants experienced virological failure. Calcagno *et al*. have shown that valproic acid administered concurrently with DTG led to lower DTG concentrations [2]. The lower concentrations were proposed to have been caused either by decreased drug absorption due to the presence of divalent cations or the induction of CYP3A4 [2]. Since this was an observational study, we did not measure DTG blood levels, instead, the impact of sodium valproate on DTG was assessed by changes in virological outcomes. No safety or efficacy concerns were noted.

For the one study participant that used St. John´s wort, the length of time that it was taken was not determined. However, the participant did have comorbidities and had undetectable VLs before and after DTG. The participant did not experience side effects, and neither was any treatment discontinuation noted in this participant. Whilst St John´s wort is associated with decreased DTG concentrations [23-25], we did not note any safety concerns with the study participant. There is also a dearth of literature documenting the effect of DTG and St John´s wort. Co-administration of aluminium and magnesium-containing antacids; and calcium and iron supplements, reduce the bioavailability of DTG by approximately 3 times [22]. In this study a total of 136 (29.5%) participants used either calcium, iron, aluminium or magnesium, alone or in combination, either in the form of supplements or antacids. Of these participants, 71 (52.2%) of them also had comorbidities, and 74 (54.4%) experienced side effects. There were 3 (2.2%) side effect-related discontinuations in this group. These side effects experienced were malaise, arthralgia, neuralgia, and dizziness. These 3 participants had more than one comorbidity and undetectable VLs prior to DTG. VLs after DTG initiation were not available as data collection was terminated when the participant discontinued DTG-based ART. Further assessments were done on the administration of calcium supplements, iron supplements, and multivitamins together with DTG. It was noted that participants were not taking these as per recommended guidance of 2 hours prior to or 6 hours after DTG intake [16,22]. Individuals taking DTG-based ART require intensive counselling on dosage timing with other non-ART drugs and supplements, to mitigate the impact of loss in efficacy due to potential drug interactions.

**Limitations:** the major challenge experienced was the missing clinical data from the medical records. In certain instances, drugs used for the treatment of comorbidities not dispensed at the HIV clinic were not documented in the medical records. Although we asked participants about the use of cation-containing drugs/supplements in relationship to their DTG dosing, we did not ask them whether they received specific counselling at the HIV clinic on the dosing of these in relation to DTG.

## Conclusion

The use of concomitant medication as a result of comorbidities, co-infection, and other medical conditions did not have an overall impact on the safety and efficacy of DTG-based ART, with only 3 (0.7%) participants discontinuing their treatment. Study participants taking rifampicin received an additional dose of DTG. Further data needs to be collected to confirm the relationship between DTG and hyperglycemia in participants taking metformin. Greater emphasis should be placed on counselling and the timing of doses in relation to DTG.

### What is known about this topic

- *There is an increased prevalence of comorbidities amongst people living with HIV/AIDS;*
- *Co-administration of DTG and some ART and non-ART drugs can lead to toxicity or reduced efficacy*.

### What this study adds

- *Use of DTG was not shown to have any causal relationship to the onset of hypertension or diabetes in the study population;*
- *There were no safety or efficacy concerns in the study population who took concomitant non-ART drugs known to interact with DTG*.
